# Significance of Umbilical Cord Leptin Profile during Pregnancy in Gestational Diabetes Mellitus—A Systematic Review and Meta-Analysis

**DOI:** 10.3390/jcm12144756

**Published:** 2023-07-18

**Authors:** María del Mar Roca-Rodríguez, Pablo Ramos-García, Cristina López-Tinoco, Manuel Aguilar-Diosdado

**Affiliations:** 1 Department of Endocrinology and Nutrition and Biomedical Research and Innovation Institute of Cadiz (INiBICA), Puerta del Mar University Hospital, 11009 Cadiz, Spain; cristinalopeztinoco@gmail.com (C.L.-T.); manuel.aguilar.sspa@juntadeandalucia.es (M.A.-D.); 2 Department of Oral Medicine, School of Dentistry, University of Granada, 18071 Granada, Spain; 3 Department of Medicine, Cadiz University (UCA), 11003 Cadiz, Spain

**Keywords:** gestational diabetes mellitus, leptin, umbilical cord, materno-fetal outcomes, systematic review, meta-analysis

## Abstract

Background: The literature provides limited evidence of cord blood leptin levels in gestational diabetes mellitus (GDM), with contradictory and inconsistent results with respect to their possible implications for maternal, perinatal, and future complications. Methods: MEDLINE/PubMed, Embase, Scopus, and Web of Science databases were searched in order to investigate the state of evidence on the association of leptin profile in cord blood during perinatal complications in GDM. We critically assessed the risk of bias using the Newcastle-Ottawa scale. Meta-analyses were performed, and heterogeneity and publication bias were analyzed. Results: sixteen primary-level studies were included, recruiting 573 GDM and 1118 control pregnant women. Cord blood leptin levels were significantly higher in GDM participants compared to controls (standardized mean difference [SMD] = 0.59, 95% confidence intervals (CI) = 0.37 to 0.80, *p* < 0.001). All subgroups also maintained significant differences stratified by continents (Asia: SMD = 0.91, 95% CI = 0.45 to 1.37, *p* < 0.001; Europe: SMD = 0.38, 95% CI = 0.20 to 0.56, *p* < 0.001), analysis technique (ELISA: SMD = 0.70, 95% CI = 0.44 to 0.97, *p* < 0.001; RIA: SMD = 0.30, 95% CI = 0.11 to 0.49, *p* = 0.002), and sample source (plasma: SMD = 0.71, 95% CI = 0.33 to 1.09, *p* < 0.001; serum: SMD = 0.55, 95% CI = 0.34 to 0.77, *p* < 0.001). Conclusion: Cord blood leptin levels were significantly higher in GDM compared to controls. Further research is needed to clarify its role as a predictive biomarker of subsequent metabolic diseases in mothers with GDM and offspring.

## 1. Introduction

It is known that many biological parameters are modified by gestational diabetes mellitus (GDM) and could potentially be used as predictors. About 1–14% of all pregnancies are complicated with GDM, which, classically, is defined as an intolerance to carbohydrates that begins, or is first recognized, during pregnancy and resolves after delivery [1]. However, it has been years since guidelines highlighted the difference in the first trimester between GDM and overt diabetes, and in fact, the American Diabetes Association (ADA) defines GDM as hyperglycemia diagnosed in the second or third trimester of gestation [2]. GDM is associated with shoulder dystocia, prolonged labor, preterm birth, preeclampsia, and instrumental delivery as prenatal morbidity; T2DM after pregnancy in mothers; macrosomia in infants and obesity, impaired glucose tolerance, and T2DM in early adulthood [3,4,5].

Fetal growth is the result of complex interactions between the placental, fetal, and maternal environments, and is controlled by genetic, hormonal, and nutritional factors. Nevertheless, the hormonal mechanisms involved in fetal growth are not fully understood. Both GDM as well as obesity/overweight during pregnancy are risk factors for hormonal neonatal and harmful anthropometric outcomes [6,7]. Previous research reported placental and umbilical cord changes in pregnancies with GDM, including increased placental expression of neoangiogenesis and inflammation markers, which is also independently associated with maternal increased pregestational BMI and gestational weight gain [8]. Furthermore, other studies demonstrated that GDM is also responsible for changes in the umbilical cord structure; in particular, in pregnancies with GDM and macrosomia, cord size is increased and Doppler resistences are diminished compared with controls [9,10]. Cord blood analysis is used to identify potential links between intrauterine exposures and long-term outcomes. A systematic review and meta-analysis in different population groups (in the absence of major maternal diseases such as pre-existing diabetes or GDM, major neonatal abnormalities, and complications during delivery) communicated a moderately positive correlation between birth weight and umbilical cord leptin levels. In addition, statistically significant positive relationships were also found for birth length and ponderal index [11]. Cord blood leptin is associated with birth weight, as well as with fat body mass, adiposity, head circumference, ponderal index, and birth length. However, it is not known whether leptin is involved in fetal growth or if it only reflects fetal adiposity [12]. Higher leptin levels were observed in the umbilical cords of newborns whose mothers had GDM. However, the reported differences did not persist after adjustment for fat mass. It has been stated that higher leptin levels in neonates of mothers with GDM might be related to increased fetal adiposity [13,14]. In addition, maternal characteristics, such as glucose concentrations, obesity, or smoking, have been directly related to an increase in cord blood leptin concentrations. It has been proposed that fetal leptin influences the programming of hypothalamic neural networks to influence long-term adiposity. A number of studies have examined the association between cord blood leptin profile and adiposity in late infancy, while another report has suggested that this relationship changes during the child’s growth with age [15].

While the role of maternal leptin profile in the development of GDM and its maternal-fetal complications has been extensively evaluated by primary studies and higher-level of evidence-based research, such as a recent meta-analysis published by our group [16], current knowledge on cord leptin levels in GDM women is limited and has conflicting results. Based on this background, the present systematic review with meta-analysis had the objective of exploring whether there are differences in cord blood leptin levels between pregnant women with GDM and controls in order to define the state of evidence about the cord leptin profile during pregnancy in GDM, its role in the pathophysiology, and its potential use as a predictive marker for the subsequent occurrence of diabetes and other metabolic disorders in GDM mothers and infants.

## 2. Materials and Methods

This systematic review and meta-analysis strictly complied with the pertinent PRISMA reporting checklist and with the MOOSE statement, specifically designed for meta-analyses of observational studies [17,18], and was methodologically designed closely following the Cochrane Collaboration criteria [19].

### 2.1. Protocol

A methodological study protocol was a priori designed with the aim of potentially reducing the risk of bias, improving transparency, precision, and, in summary, the integrity of the present study. It was also registered in the PROSPERO international prospective register of systematic reviews (www.crd.york.ac.uk/PROSPERO (accessed and registered on 26 July 2020), CRD42020194274 code) [20]. The protocol also complied with PRISMA-P reporting guidelines to ensure a rigorous approach [21].

### 2.2. Search Strategy

MEDLINE (through PubMed), Embase, Web of Science, and Scopus databases were searched for studies published before March 2022 (upper limit), with no lower date limits. The search strategy was driven by combining pertinent thesaurus terms (i.e., EMTREE and MeSH terms) with free terms, and built to maximize sensitivity. The following syntax was implemented in MEDLINE and adapted for the rest of databases (Supplementary Table S1): (“Diabetes, Gestational”[MeSH Terms] OR “Gestational diabetes”[All Fields] OR “Pregnancy in Diabetics”[MeSH Terms] OR “pregnancy diabetes mellitus”[All Fields] OR “GDM”[All Fields]) AND (“leptin”[MeSH Terms] OR “leptin”[All Fields]). Handsearching methods were also implemented, screening the reference lists of target studies in order to search for additional primary-level studies. Study references were processed using a specific software (Mendeley Desktop v.1.19.8, Elsevier, Amsterdam, The Netherlands); duplicates were detected and removed through this software.

### 2.3. Eligibility Criteria

Inclusion criteria: (1) Primary-level studies without publication date or language restrictions, or without follow-up, geographical areas, or patients’ age limits; (2) GDM patients were compared to controls (non-GDM pregnant women); (3) Cord plasma or serum leptin profile levels assessment; (4) Observational studies, regardless of their prospective/retrospective nature or their cross-sectional/longitudinal design.

Exclusion criteria: (1) Withdrawal papers, interventional studies, meta-analyses, reviews, letters, editorials, meeting abstracts, personal comments, book chapters, and case reports; (2) Preclinical studies (animal experimentation or *in vitro*); (3) Studies that do not investigate the disease of interest (i.e., GDM), do not study cord leptin profile, or without a control group; (4) Insufficient essential data to analyze means ± standard deviations (SD); (5) Data from overlapping populations.

### 2.4. Study Selection Process

Eligibility criteria were applied independently by two authors (M.d.M.R.-R. and C.L.-T.). Discrepancies among both researchers were resolved by consensus with a third author (P.R.-G.). Articles were selected in two stages: first, piloting the titles with abstracts of the retrieved registers; and second, full-text reading the potential sample, excluding those that did not really meet the review eligibility criteria.

### 2.5. Data Extraction

Two authors (M.d.M.R.-R. and C.L.-T.) separately extracted the datasets of interest from the chosen articles by using Excel software (version Professional Plus 2013, Microsoft, Redmond, WA, USA). Data were gathered on the study authors, year of publication, geographical area, publication language, sample size, study design, source of sample (serum/plasma), cord leptin profile determination (means ± SD, measuring units, proper quantification, and technique) in GDM and controls, diagnostic criteria for GDM, criteria for control sampling, personal and family risk of diabetes, gestational age, control of risk factors during pregnancy (maternal age, gestational and pregestational body mass index [BMI], glucose, insulin, homeostasis model assessment of insulin resistance (HOMA-IR), glycosylated hemoglobin (A1cHb), fetal and maternal outcomes, follow-up period, and assessment of patient drop-out rate.

### 2.6. Evaluation of Quality and Risk of Bias

The Newcastle-Ottawa quality assessment scale (NOS) was used in order to assess the risk of bias (RoB) [22]. RoB critical appraisal was performed by two independent reviewers with methodological and content expertise (M.d.M.R.-R. and C.L.-T.). The results were compared, and conflicts were resolved by agreement between the two reviewers, with the input of a third reviewer (P.R.-G.) if required. The maximum score was 9, and the minimum was 0. A score equal to or higher than 8 was a priori set as a sign of high methodological quality (i.e., low RoB), a score of 7 or 6 was considered moderate quality (i.e., moderate RoB), and a score equal to or lower than 5 was judged as low quality (i.e., high RoB).

### 2.7. Statistical Analysis

Mean ± SD cord blood leptin profile levels were managed from the study sample in order to contrast the GDM group vs. controls. Standardized mean differences (SMDs)—estimated by Cohen’s d method—with 95% confidence intervals (CI) were computed and adopted as effect size metrics based on the expected methodological heterogeneity, mainly due to variations in leptin determination methods and heterogeneous laboratory protocols. If only medians with an interquartile range and/or maximum-minimum ranges were available, means ± SDs were estimated following Luo et al. [23] and Wan et al. [24] methods. The Cochrane formula was also applied if it was desirable to combine means ± SD from different subgroups into a single group [19]. When data were only graphically expressed, data extraction was conducted through the use of a specific digitizing software (Engauge-Digitizer version 4.1, open-source developed by M. Mitchell). SMDs and 95% Cis were meta-analyzed applying the inverse-variance method under a random-effects model (based on the DerSimonian and Laird method), which accounts for the possibility that there are different underlying results among study subpopulations (i.e., related to the heterogeneity inherent in the wide range of experimental methods, leptin profile level variations among cord blood plasma and serum, etc.). Forest plots were plotted in order to graphically represent the meta-analytical overall effects and for subsequent visual inspection analyses (significant differences were set at *p* < 0.05). Statistical heterogeneity was assessed using the Cochran’s Q test (a *p*-value < 0.10 was considered significant due to the low statistical power of this test) and quantified through the I^2^ statistic (25–50–75% values were interpreted as low-to-moderate-to-high degree of inconsistency), which estimates what proportion of the variance in observed effects reflects variation in true effects rather than sampling error [25,26]. Preplanned stratifications and univariable meta-regression analyses by methodological and clinico-analytical variables of interest in order to explore potential sources of heterogeneity [27]. Weighted bubble plots with fitted meta-regression lines were also plotted for illustrative purposes. Sensitivity analyses were also carried out in order to test the reliability of the meta-analytical result by applying the canonical “leave-one-out” method). Finally, the analysis of small study effects, such as publication bias, was conducted via the Egger regression test (*p* < 0.10 considered significant) and funnel plot construction [28,29]. Stata version 16.1 (Stata Corp., College Station, TX, USA) was employed for all tests during the statistical analysis [30].

## 3. Results

### 3.1. Results of the Literature Search

**Figure 1:**
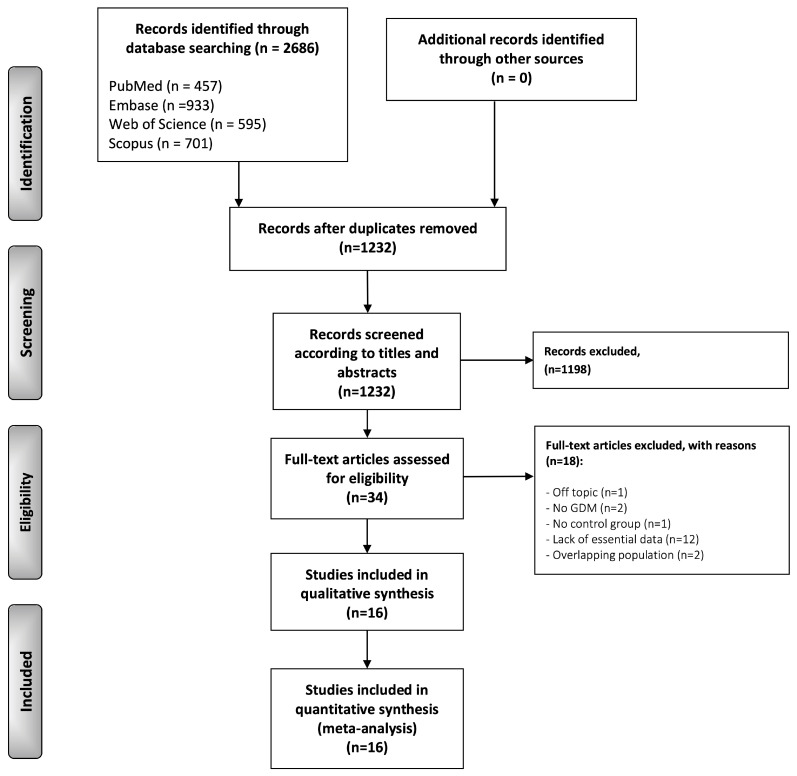
Flow diagram. Identification and selection process of relevant studies comparing cord blood leptin levels between GDM patients and controls. Study characteristics.

The flow diagram in Figure 1 depicts the process of identification and selection of targeted primary-level studies. We retrieved a total of 2686 records published before March 2022: 457 from MEDLINE/PubMed, 933 from Embase, 595 from the Web of Science, and 701 from Scopus. After eliminating duplicates, 1232 studies were considered potentially eligible. After screening their titles and abstracts, 34 were selected for full-text reading (full-text articles were excluded, and their exclusion reasons were listed in the Supplementary Information). Finally, after the exclusion of the studies that did not meet all eligibility criteria, 16 studies were included in our systematic review and meta-analysis.

The characteristics of our study sample (*n* = 16 primary-level studies) are summarized in Table 1 and Table S2, which describe in more detail the parameters managed in each study. Our studies compare the changes in cord leptin profile levels in a total of 1691 participants (573 GDM patients vs. 1118 controls). Cord blood serum was analyzed in 9 studies, while cord blood plasma was analyzed in 6 studies, and only a single study did not specify it. Leptin was determined and quantified by enzyme-linked immunosorbent assay (ELISA) in 10 studies and by radioimmunoassay (RIA) in 6 studies. Subsample sizes ranged between 6 and 327 women across studies.

### 3.2. Qualitative Evaluation

The qualitative analysis was conducted using the NOS RoB Scale, which evaluates potential sources of bias in nine domains (Table 2).

This systematic review only included studies with GDM patients and controls appropriately selected and matched. Studies without a control group were directly excluded. Primary-level studies were categorized as low (37.5%), moderate (37.5%), and high (25%) overall RoB. The most frequent potential biases were an inadequate description of fetal or maternal outcomes and the failure to report follow-up period information. In this regard, the RoB with respect to the follow-up and drop-out rates was elevated in 81.3% of the studies. It is worth highlighting the relevance of declaring the lost to the follow-up, which is essential data to evaluate any differences in obstetric and perinatal outcomes and on the subsequent follow-up and development of complications in both the child and the mother.

### 3.3. Quantitative Evaluation (Meta-Analysis)

**Figure 2:**
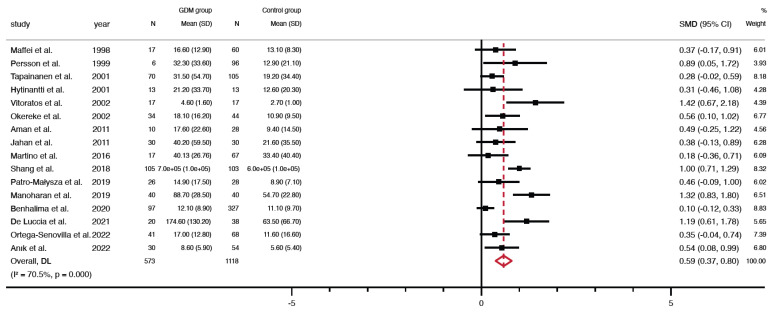
Forest plots graphically represent the meta-analysis evaluating the changes in cord blood leptin levels between GDM patients and controls (random-effects model, inverse-variance weighting based on the DerSimonian and Laird method). Standardized mean difference (SMD) was chosen as the effect size measure. A SMD > 0 suggests that cord blood leptin levels are higher in GDM. The diamond indicates the overall pooled SMDs with their corresponding 95% confidence intervals (CI) [xx,xx,xx].

Meta-analysis on cord blood leptin in GDM. Levels were significantly higher in GDM participants than in controls (SMD = 0.59, 95% CI = 0.37 to 0.80, *p* < 0.001), showing significant heterogeneity (*p* < 0.001, I^2^ = 70.5%; Figure 2, Table 3).

Analysis of subgroups. All subgroups maintained the precedent significant differences after subgroup meta-analyses were carried out (Table 3, Figures S1–S4), stratified by geographical area (Asia: SMD = 0.91, 95%CI = 0.45 to 1.37, *p* < 0.001; Europe: SMD = 0.38, 95%CI = 0.20 to 0.56, *p* < 0.001), by analysis technique (ELISA: SMD = 0.70, 95%CI = 0.44 to 0.97, *p* < 0.001; RIA: SMD = 0.30, 95%CI = 0.11 to 0.49, *p* = 0.002), by sample source (cord blood plasma: SMD = 0.71, 95%CI = 0.33 to 1.09, *p* < 0.001; cord blood serum: SMD = 0.55, 95%CI = 0.34 to 0.77, *p* < 0.001). Finally, the primary-level studies showing a low risk of bias also maintained this significant difference and harbored a large effect size (SMD = 0.93, 95%CI = 0.57 to 1.30, *p* < 0.001).

Univariable meta-regression analyses. The effect of the remaining study covariates on cord blood leptin levels among patients with GDM compared to controls showed no significant differences (Table 4, Figures S5–S13).

### 3.4. Quantitative Evaluation (Secondary Analyses)

Sensitivity analysis. The precedent overall results did not substantially vary after the sequential repetition of meta-analyses, omitting one study each time (Figure S14). This suggests that the combined estimations reported do not depend on the influence of a particular individual primary-level study.

Small-study effects analysis. The visual inspection analysis of the asymmetry of the funnel plot constructed (Figure S15) and the statistical test conducted for the same purpose (p_Egger_ = 0.02) revealed the presence of small-study effects, thus publication bias could not be potentially ruled out.

## 4. Discussion

This systematic review with meta-analysis has included 16 primary-level studies and 1691 patients. It is shown that cord leptin profile levels were significantly higher in women with GDM than in the control group (SMD = 0.59, 95% CI = 0.37 to 0.80, *p* < 0.001). This finding is consistent with previous studies in this regard [15,31,32,33,34], in contrast with the absence of differences objectified by other authors [35,36,37]. On the other hand, lower cord leptin levels than maternal levels, with no differences between women with and without GDM, have been found by Mosavat M et al. [38]. Regarding cord leptin levels, Johnson AW et al. [39], assessing obese mothers and T2DM/GDM, have observed significantly lower cord venous and cord arterial to maternal plasma ratios of insulin but not leptin compared with lean mothers. However, these authors have reported no differences in cord blood insulin and leptin levels between obese and diabetic mothers [39].

In our meta-regression analyses, significant differences were not found among the study covariates on cord blood leptin profile levels across GDM patients compared to the control group. This fact is probably due to the limited number of studies analyzing these variables. Similar results have been reported by Shekhawat PS et al. [40], showing no impact of maternal obesity on cord blood leptin. Likewise, Wang WJ et al. [36] have found no statistically significant differences between insulin, proinsulin, or C-peptide in cord blood. While Ott R et al. [41] showed that certain pre-pregnancy maternal anthropometric parameters (weight, BMI, gestational weight gain) were significant factors for elevated cord blood insulin and leptin levels, these results lost statistical significance after adjustment for maternal glucose during late pregnancy. Nevertheless, some authors have detected significant differences among clinical-analytical variables of interest. A significant association between cord leptin levels and maternal BMI has been reported between mothers with [15,34,42] and without GDM [43]. Shang et al. [32] have communicated that cord plasma leptin levels are inversely correlated with HOMA-IR in women with GDM. On the contrary, Manoharan B et al. [33] have found higher levels of cord plasma HOMA-IR, insulin, and C-peptide in GDM and a significant positive correlation between cord plasma HOMA-IR and the leptin/adiponectin ratio. On the other hand, Niknam A et al. [44] have observed a significant correlation between C-peptide cord concentrations and the incidence of maternal GDM and neonatal macrosomia. Furthermore, Ortega-Senovilla H et al. [37] have also reported higher cord serum glucose and insulin levels in GDM than in controls.

Some authors have also described a significant and positive correlation between neonatal birth weight/ponderal index in normal pregnancy and cord leptin profile [31,40,45]. In this sense, a systematic review and meta-analysis [11] have confirmed that cord blood leptin levels are positively associated with birthweight, explaining one fifth of the variation in birthweight in the population. This finding was consistent between males and females, as well as between Caucasian and Asian populations. While a lower effect was also observed for small for gestational age (SGA) neonates compared to appropriate for gestational age neonates (AGA). This outcome occurred in the absence of specific referral to complications during delivery, major neonatal abnormalities, or maternal diseases, including pre-existing or GDM [11]. Kang SJ et al. [46] have observed that third trimester cord leptin levels were higher with increasing gestational age, which was independently associated with fetal growth. The log cord serum leptin profile was independently associated with length and birth weight in multivariable linear regression analysis after adjustment for potentially confounding factors. Also, in GDM, cord blood leptin levels have been positively correlated with birth weight [34,42]. Higher levels were present in macrosomia compared to normal birth weight newborns [32], while lower levels were present in SGA infants with either stunting only or wasting only [47]. Furthermore, a positive association between cord blood adipokines and adiposity in early childhood has recently been described [15]. Another recent study randomized women with GDM in the TARGET trial to strict or less strict glycemic targets. It has been concluded that the use of tighter glycemic targets does not result in substantial changes in maternal or cord plasma biomarkers. However, maternal serum leptin and infant cord C-peptide and leptin results were lower for women who complied with tighter targets. However, maternal serum leptin, infant C-peptide, and cord leptin levels were lower in women who met stricter targets [48].

According to our risk of bias (RoB) analysis, which was carried out using the Newcastle-Ottawa Quality Assessment Scale (NOS tool), all the studies included in our systematic review were not methodologically designed with the same rigor. A meta-analysis stratified by overall risk of bias revealed that the subgroup of studies designed with better methodological quality (i.e., lower risk of bias) achieved better meta-analytic performance. (SMD = 0.93, 95%CI = 0.57 to 1.30, *p* < 0.001). It means that these higher-quality primary-level studies identified the highest differences in cord blood leptin levels between pregnant women with GDM and controls. This fact increases the quality of the evidence of the results reported in our meta-analysis, which could even be underestimated in the studies reporting a small effect size and harboring a higher risk of bias. Therefore, future studies focused on this topic should consider the potential biases and recommendations reported in this systematic review and meta-analysis to improve and standardize future research.

To the best of our knowledge, this is the first systematic review and meta-analysis about cord leptin levels in GDM. A potential limitation of our meta-analysis is the existence of a moderate degree of statistical heterogeneity. It should be noted that clinical and methodological heterogeneity was expected, so random-effects models were *a priori* planned and performed for all meta-analyses. In addition, more homogeneous subgroups were observed after stratified meta-analyses, with some subgroups yielding consistent non-heterogeneous results. Another possible limitation is the suspicion of publication bias, which could not be ruled out in this meta-analysis. The current tendency to publish positive results as a priority is an inherent obstacle to the editorial process that is difficult to overcome in biomedicine. Regardless, the SMD reported in our meta-analysis for cord leptin levels was considerably higher in GDM versus controls, and the sensitivity analyses performed without relevant variation in the magnitude of the effect support, in summary, the stability and robustness of our results.

## 5. Conclusions

Our meta-analysis evidences higher cord blood leptin levels in women with GDM compared to controls. However, given the few papers reporting clinical-metabolic variables, a more comprehensive analysis of the possible involvement of cord blood leptin in the pathophysiology of GDM and maternal-fetal outcomes in these patients has not been possible. Thus, it is not yet known the possible importance of higher levels of cord leptin in maternal-fetal outcomes. The limitations of the current methods of fetal anthropometric assessment or prevention strategy for GDM demonstrate the need to discover early predictors of GDM to establish intervention and prevention strategies in high-risk women. Further studies are needed to evaluate the role of cord leptin and other biomolecule levels in the future development of T2DM and other metabolic diseases in GDM mothers and offspring.

## Figures and Tables

**Table 1:** Summarized characteristics of reviewed studies.

Total	16 studies
Year of publication	1998–2022
Number of patients	
Total	1691 patients
Cases with GDM	573 patients
Controls	1118 patients
Sample size, range	6–327 patients
Leptin determination	
ELISA	10 studies
RIA	6 studies
Source of samples	
Serum	9 studies
Plasma	6 studies
Serum or plasma not specified	1 studies
Geographical region	
Europe	10 studies
Asia	3 studies
Europe–Asia	1 study
North America	1 study
South America	1 study

**Table 2:** Summary of risk of bias assessment based on the Newcastle-Ottawa Quality Assessment Scale. Two reviewers who had content and methodological expertise assessed and graded the risk of bias for the included studies, independently and in duplicate, with an adapted version of the Newcastle-Ottawa scale (NOS), which has been described elsewhere. The assessments were compared, and conflicts were resolved by agreement between the two reviewers. The maximum score was eight, and the minimum score was zero. It was decided a priori that a score of seven was reflective of high methodological quality (e.g., low risk of bias), a score of five or six indicated moderate quality, and a score of four or less indicated low quality (e.g., high risk of bias). A filled blue star indicates that a star has been awarded, and a blank star indicates that no star has been awarded, and the study has been graded as poor quality in that category. Overall risk of bias (RoB): 8–9 low, 6–7 moderated, ≤5 high.

Study	Selection			Control		Outcomes				Total Score	Overall RoB
	Selection of GDM Patients	Selection of Non-GDM Subjects	Family/ Personal GDM Risk Factors	Control of Risk Factors during Pregnancy	Properly Leptin Quantification	Maternal Outcomes	Fetal Outcomes	Appropriate Follow-up Period	Adequacy of Follow-up		
**Maffei et al., 1998**	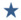	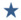	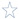	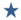	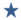	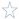	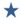	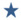	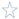	6	
**Persson et al., 1999**	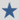	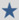	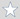	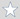	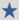	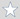	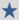	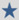	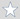	5	
**Tapainanen et al., 2001**	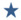	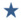	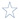	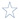	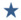	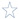	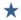	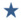	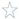	5	
**Hytinantti et al., 2001**	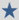	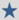	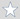	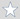	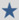	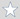	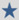	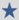	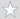	5	
**Vitoratos et al., 2002**	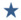	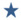	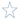	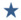	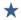	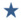	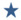	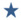	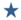	8	
**Okereke et al., 2002**	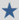	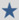	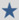	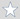	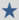	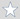	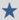	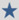	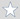	6	
**Aman et al., 2011**	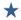	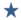	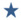	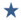	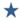	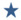	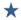	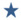	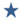	9	
**Jahan et al., 2011**	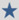	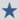	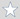	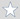	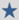	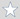	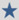	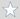	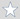	4	
**Martino et al., 2016**	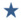	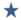	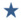	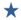	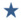	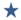	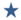	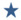	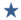	9	
**Shang et al., 2018**	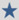	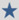	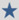	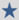	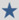	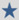	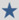	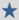	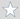	8	
**Patro-Małysza et al., 2019**	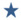	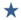	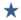	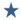	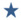	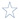	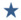	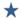	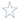	7	
**Manoharan et al., 2019**	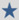	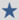	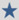	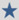	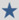	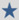	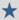	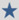	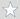	8	
**Benhalima et al., 2020**	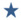	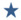	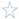	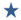	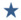	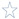	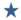	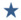	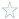	6	
**De Luccia et al., 2021**	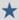	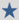	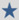	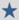	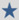	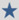	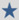	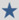	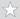	8	
**Ortega-Senovilla et al., 2022**	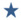	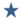	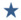	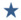	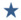	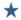	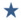	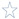	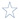	7	
**Anık et al., 2022**	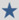	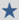	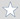	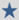	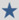	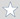	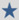	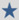	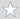	6	

**Table 3:** Meta-analysis of cord blood leptin levels in GDM.

					Pooled Data		Heterogeneity	
Meta-Analyses	No. of Studies	No. of Patients	Stat. Model	Wt	SMD (95% CI)	*p*-Value	*P_het_*	I^2^ (%)
All ^a^	16	1691	REM	D-L	0.59 (0.37 to 0.80)	<0.001	<0.001	70.5
Subgroup analysis by continent ^b^								
Asia	3	348	REM	D-L	0.91 (0.45 to 1.37)	<0.001	0.03	71.9
Europe	11	1207	REM	D-L	0.38 (0.20 to 0.56)	<0.001	0.13	33.8
North America	1	78	—	—	0.56 (0.10 to 1.02)	0.02	—	—
South America	1	58	—	—	1.19 (0.61 to 1.78)	<0.001	—	—
Subgroup analysis by analysis technique ^b^								
ELISA	10	848	REM	D-L	0.70 (0.44 to 0.97)	<0.001	0.001	67.3
RIA	6	843	REM	D-L	0.30 (0.11 to 0.49)	0.002	0.30	17.0
Subgroup analysis by sample source ^b^								
Plasma	6	577	REM	D-L	0.71 (0.33 to 1.09)	<0.001	0.008	67.7
Serum	9	690	REM	D-L	0.55 (0.34 to 0.77)	<0.001	0.08	42.7
Not reported	1	424	—	—	0.10 (−0.12 to 0.33)	0.36	—	—
Subgroup analysis by study design ^b^								
Prospective	16	1691	REM	D-L	0.59 (0.37 to 0.80)	<0.001	<0.001	70.5
Retrospective	0	0	—	—	—	—	—	—
Subgroup analysis by RoB ^b^								
High RoB	4	363	REM	D-L	0.35 (0.12 to 0.59)	0.003	0.61	0.0
Moderate RoB	6	826	REM	D-L	0.31 (0.14 to 0.48)	<0.001	0.35	10.6
Low RoB	6	502	REM	D-L	0.93 (0.57 to 1.30)	<0.001	0.02	64.6

Abbreviations: Stat., statistical; Wt, method of weighting; SMD, standardized mean difference; CI, confidence intervals; REM, random-effects model; D-L, DerSimonian and Laird method; GDM, gestational diabetes mellitus. ^a^—Meta-analysis; ^b^—Subgroup meta-analysis.

**Table 4:** Meta-regression analysis.

Covariate	No. of Studies	No. of Patients	Stat. Model	Coef. (95% CI)	*p*-Value	Heterogeneity Explained
Maternal age in GDM (years)	11	1250	random-effects meta-regression	−0.046 (−0.145 to 0.054) ^a^	0.33 ± 0.005 ^b^	5.25% ^c^
Pregestational BMI in GDM (summary index score)	9	742	random-effects meta-regression	−0.073 (−0.257 to 0.110) ^a^	0.42 ± 0.005 ^b^	−1.54% ^c^
Gestational BMI in GDM (summary index score)	2	163	—	—	—	—
Maternal glycemia levels in GDM (mmol/L)	5	902	random-effects meta-regression	−0.099 (−1.202 to 1.399) ^a^	0.79 ± 0.004 ^b^	−27.50% ^c^
Maternal insulin in GDM (pmol/L)	3	351	—	—	—	—
Maternal HbA1c in GDM (%)	4	763	—	—	—	—
Maternal HOMA in GDM summary index score)	2	632	—	—	—	—
Cord blood glycemia levels in GDM (mmol/L)	4	354	—	—	—	—
Cord blood Insulin in GDM (mcU/mL)	6	539	random-effects meta-regression	−0.023 (−0.065 to 0.019) ^a^	0.15 ± 0.004 ^b^	0.00% ^c^
Cord blood C peptide in GDM (nmol/L)	5	701	random-effects meta-regression	−0.421 (−0.803 to 1.644) ^a^	0.45 ± 0.005 ^b^	24.92% ^c^
Gestational age delivery in GDM (weeks)	14	1522	random-effects meta-regression	−0.251 (−0.615 to 0.112) ^a^	0.17 ± 0.004 ^b^	8.96% ^c^
Caesarian in GDM (%)	7	611	random-effects meta-regression	0.005 (−0.012 to 0.021) ^a^	0.50 ± 0.005 ^b^	−5.59% ^c^
Newborn weight in GDM (gr)	16	1691	random-effects meta-regression	−0.0004 (−0.0015 to 0.0006) ^a^	0.41 ± 0.005 ^b^	−2.62% ^c^
Macrosomy in GDM (%)	5	873	random-effects meta-regression	0.0001 (−0.0666 to 0.0670) ^a^	0.99 ± 0.005 ^b^	−38.53% ^c^

Abbreviations: Stat., statistical; coef, coefficient; CI, confidence intervals; GDM, gestational diabetes mellitus. ^a^—Meta-regression coefficient on the effect of study covariates on cord blood leptin levels among patients with GDM compared with controls. A meta-regression coefficient > 0 indicates a greater impact of covariates on effect size. ^b^—*p*-value ± standard error after 10,000 permutations based on Montecarlo simulation. ^c^—Proportion of between-study variance explained (adjusted R2 statistic), expressed as a percentage, using the residual maximum likelihood (REML) method. A negative proportion reflects no heterogeneity.

## Data Availability

The search strategy, study selection process, data extraction, and analysis were detailed in the materials and methods and in Supplementary Information to the manuscript.

## Supplementary Materials

The following supporting information can be downloaded at: https://www.mdpi.com/article/10.3390/jcm12144756/s1.
