# Supplementary material for: Significance of Umbilical Cord Leptin Profile during Pregnancy in Gestational Diabetes Mellitus—A Systematic Review and Meta-Analysis
Source: J Clin Med. 2023 Jul 18;12(14):4756. doi: 10.3390/jcm12144756 (PMC10381212; doi:10.3390/jcm12144756)
Supplement: Supplementary file 1 [file jcm-12-04756-s001.zip › jcm-2410224-supplementary.pdf]

Supplementary Information to the manuscript

Significance of Umbilical Cord Leptin Profile during Pregnancy in Gestational Diabetes Mellitus—A Systematic Review and Meta-Analysis

María del Mar Roca-Rodríguez [1,*,†], Pablo Ramos-García [2,*,†], Cristina López-Tinoco [1,3] and Manuel Aguilar-Diosdado [1,3]

[1] Department of Endocrinology and Nutrition and Biomedical Research and Innovation Institute of Cadiz (INiBICA), Puerta del Mar University Hospital, 11009 Cadiz, Spain; cristinalopeztinoco@gmail.com (C.L.-T.); manuel.aguilar.sspa@juntadeandalucia.es (M.A.-D.)

[2] Department of Oral Medicine, School of Dentistry, University of Granada, 18071 Granada, Spain

[3] Department of Medicine, Cadiz University (UCA), 11003 Cadiz, Spain

* Correspondence: maroca80@gmail.com (M.d.M.R.-R.); pabloramos@ugr.es (P.R.-G.)

† These authors contributed equally to this work.

1. Search strategy

Table S1. Search strategy for each database, number of results, and execution date.

Database	Query	Results	Upper date limit
PubMed	(“Diabetes, Gestational”[MeSH Terms] OR “Gestational diabetes”[All Fields] OR “Pregnancy in Diabetics”[MeSH Terms] OR “pregnancy diabetes mellitus”[All Fields] OR “GDM”[All Fields]) AND ("leptin"[MeSH Terms] OR "leptin"[All Fields])	457	March, 2022
Embase	('pregnancy diabetes mellitus'/exp OR 'pregnancy diabetes mellitus' OR 'Gestational diabetes' OR 'GDM') AND ('leptin'/exp OR 'leptin')	933	March, 2022
Web of Science	TS=("Gestational diabetes" OR "pregnancy diabetes mellitus" OR "GDM") AND TS=("leptin")	595	March, 2022
Scopus	TITLE-ABS-KEY((“Gestational diabetes” OR “pregnancy diabetes mellitus” OR “GDM”) AND “leptin”)	701	March, 2022
Total			2686

2. Full-text articles excluded (n = 18)

Marchini G, Fried G, Ostlund E, Hagenäs L. Plasma leptin in infants: relations to birth weight and weight loss. Pediatrics. 1998 Mar;101(3 Pt 1):429-32. doi: 10.1542/peds.101.3.429. PMID: 9481009.

No GDM

Gross GA, Solenberger T, Philpott T, Holcomb WL Jr, Landt M. Plasma leptin concentrations in newborns of diabetic and nondiabetic mothers. Am J Perinatol. 1998 Apr;15(4):243-7. doi: 10.1055/s-2007-993935. PMID: 9565222.

Lack of essential data

Lepercq J, Cauzac M, Lahlou N, Timsit J, Girard J, Auwerx J, Hauguel-de Mouzon S. Overexpression of placental leptin in diabetic pregnancy: a critical role for insulin. Diabetes. 1998 May;47(5):847-50. doi: 10.2337/diabetes.47.5.847. PMID: 9588462.

Lack of essential data

Lewandowski K, Horn R, O'Callaghan CJ, Dunlop D, Medley GF, O'Hare P, Brabant G. Free leptin, bound leptin, and soluble leptin receptor in normal and diabetic pregnancies. J Clin Endocrinol Metab. 1999 Jan;84(1):300-6. doi: 10.1210/jcem.84.1.5401. PMID: 9920099.

No GDM

Silva NY, Tennekoon KH, Senanayake L, Karunanayake EH. Cord blood leptin levels in normal pregnancies, pregnancy induced hypertension and gestational diabetes mellitus. Ceylon Med J. 2008 Sep;53(3):79-82. doi: 10.4038/cmj.v53i3.246. PMID: 18982799.

Lack of essential data

Jahan S, Zinnat R, Hassan Z, Biswas KB, Habib SH. Gender differences in serum leptin concentrations from umbilical cord blood of newborn infants born to nondiabetic, gestational diabetic and type-2 diabetic mothers. Int J Diabetes Dev Ctries. 2009 Oct;29(4):155-8. doi: 10.4103/0973-3930.57346. PMID: 20336197; PMCID: PMC2839129.

Overlapping population

Parker M, Rifas-Shiman SL, Belfort MB, Taveras EM, Oken E, Mantzoros C, Gillman MW. Gestational glucose tolerance and cord blood leptin levels predict slower weight gain in early infancy. J Pediatr. 2011 Feb;158(2):227-33. doi: 10.1016/j.jpeds.2010.07.052. Epub 2010 Sep 19. PMID: 20855080; PMCID: PMC4270123.

Lack of essential data

Ortega-Senovilla H, Schaefer-Graf U, Meitzner K, Abou-Dakn M, Graf K, Kintscher U, Herrera E. Gestational diabetes mellitus causes changes in the concentrations of adipocyte fatty acid-binding protein and other adipocytokines in cord blood. Diabetes Care. 2011 Sep;34(9):2061-6. doi: 10.2337/dc11-0715. Epub 2011 Jul 20. PMID: 21775757; PMCID: PMC3161255.

Overlapping population

Vela-Huerta MM, Amador-Licona N, Anaya-Aguirre S, Guizar-Mendoza JM, Velazquez-Bustamante A, Murillo-Ortiz B. Insulin and Leptin Levels in Appropriate-for-Gestational-Age Infants of Diabetic Mother. Iran J Pediatr. 2012 Dec;22(4):475-80. PMID: 23429837; PMCID: PMC3533147.

Lack of essential data

Kara M, Orbak Z, Döneray H, Ozkan B, Akcay F. The Relationship Between Skinfold Thickness and Leptin, Ghrelin, Adiponectin, and Resistin Levels in Infants of Diabetic Mothers. Fetal Pediatr Pathol. 2017 Feb;36(1):1-7. doi: 10.1080/15513815.2016.1217960. Epub 2016 Aug 25. PMID: 27559858.

Lack of essential data

Al-Daghri NM, Al-Hazmi HA, Al-Ajlan A, Masoud MS, Al-Amro A, Al-Ghamdi A, Alnaami AM, Al-Attas OS, Alokail MS. Associations of Spexin and cardiometabolic parameters among women with and without gestational diabetes mellitus. Saudi J Biol Sci. 2018 May;25(4):710-714. doi: 10.1016/j.sjbs.2018.01.002. Epub 2018 Jan 20. PMID: 29740234; PMCID: PMC5936879.

Off topic

Shapiro GD, Arbuckle TE, Ashley-Martin J, Fraser WD, Fisher M, Bouchard MF, Monnier P, Morisset AS, Ettinger AS, Dodds L. Associations between maternal triclosan concentrations in early pregnancy and gestational diabetes mellitus, impaired glucose tolerance, gestational weight gain and fetal markers of metabolic function. Environ Res. 2018 Feb;161:554-561. doi: 10.1016/j.envres.2017.12.001. PMID: 29241065.

Lack of essential data

Ott R, Stupin JH, Loui A, Eilers E, Melchior K, Rancourt RC, Schellong K, Ziska T, Dudenhausen JW, Henrich W, Plagemann A. Maternal overweight is not an independent risk factor for increased birth weight, leptin and insulin in newborns of gestational diabetic women: observations from the prospective 'EaCH' cohort study. BMC Pregnancy Childbirth. 2018 Jun 20;18(1):250. doi: 10.1186/s12884-018-1889-8. PMID: 29925339; PMCID: PMC6011392.

No control group

Kang SJ, Bae JG, Kim S, Park JH. Birth anthropometry and cord blood leptin in Korean appropriate-for-gestational-age infants born at ≥ 28 weeks' gestation: a cross sectional study. Int J Pediatr Endocrinol. 2020;2020:12. doi: 10.1186/s13633-020-00082-6. Epub 2020 Jun 26. PMID: 32607107; PMCID: PMC7318406.

Lack of essential data

Johnson AW, Snegovskikh D, Parikh L, DeAguiar RB, Han CS, Hwang JJ. Characterizing the Effects of Diabetes and Obesity on Insulin and Leptin Levels amongst Pregnant Women. Am J Perinatol. 2020 Sep;37(11):1094-1101. doi: 10.1055/s-0040-1702988. Epub 2020 Mar 2. PMID: 32120424.

Lack of essential data

Mitanchez D, Jacqueminet S, Lebbah S, Dommergues M, Hajage D, Ciangura C. Relative Contribution of Gestational Weight Gain, Gestational Diabetes, and Maternal Obesity to Neonatal Fat Mass. Nutrients. 2020 Nov 9;12(11):3434. doi: 10.3390/nu12113434. PMID: 33182482; PMCID: PMC7698189.

Lack of essential data

Tan K, Tint MT, Michael N, Yap F, Chong YS, Tan KH, Godfrey KM, Larbi A, Lee YS, Chan SY, Fortier MV, Eriksson JG, Karnani N. Determinants of cord blood adipokines and association with neonatal abdominal adipose tissue distribution. Int J Obes (Lond). 2022

Mar;46(3):637-645. doi: 10.1038/s41366-021-00975-3. Epub 2021 Dec 4. PMID: 34864815; PMCID: PMC8873009.

Lack of essential data

Fyfe R, Burton A, McLennan A, McCudden L, Gordon A, Hyett J. Factors affecting cord blood leptin levels in a consecutive birth cohort. J Matern Fetal Neonatal Med. 2022 Mar;35(5):884-889. doi: 10.1080/14767058.2020.1733518. Epub 2020 Jun 29. PMID: 32594793.

Lack of essential data

3. Table S2. Characteristics of analyzed studies (n=16).

Study	Year	Country	Continent	No. of patients with GDM	M(+/-DE) Cord Leptin GDM	No. of Patients controls	M(+/-DE) Cord Leptin controls	Technique	Units	Source of data	Sample source	Study Design	prospective/retrospective	M (+/-DE) Maternal Age GDM	M (+/-DE) Maternal Age controls	M (+/-DE) pregestational BMI GDM	M (+/-DE) pregestational BMI controls	Insulin therapy	M (+/-DE) gestational age at delivery GDM	M (+/-DE) gestational age at delivery controls	M (+/-DE) Caesarean GDM	M (+/-DE) Caesarean controls	M (+/-DE) newborn weight GDM	M (+/-DE) newborn weight controls
Maffei et al.	1998	Italy	Europe	17	16,6 ± 12,9	60	13,1 ± 8,3	ELISA	ng/ml	mean-sd	plasma	case-control	prospectivo	31,7 ± 3,2	29 ± 4,5	25,4 ± 4,1	23,3 ± 3,4	Yes	38,8 ± 2,4	39 ± 2,6	N/A	N/A	3442 ± 528	3433 ± 618
Persson et al.	1999	Sweden	Europe	6	32,3 ± 33,6	96	12,9 ± 21,1	RIA	ng/ml	mean-sd	plasma	case-control	prospectivo	N/A	N/A	N/A	N/A	Yes	37,3 ± 1,1	39,9 ± 1,1	N/A	N/A	3674 ± 637	3821 ± 428
Tapainanen et al.	2001	Finland	Europe	70	31,5 ± 54,7	105	19,2 ± 34,4	RIA	mcg/L=ng/ml	median-iqr	serum	case-control	prospectivo	N/A	N/A	N/A	N/A	N/A	39,4 ± 1,3	39,9 ± 1,5	N/A	N/A	3789 ± 598	3701 ± 513
Hytinantti et al.	2001	Finland	Europe	13	21,2 ± 33,7	13	12,6 ± 20,3	RIA	mcg/L=ng/ml	median-iqr	plasma	case-control	prospectivo	N/A	N/A	N/A	N/A	No	40,2 ± 1,4	40,3 ± 1,4	N/A	N/A	3757 ± 614	3630 ± 491
Vitoratos et al.	2002	Greece	Europe	17	4,6 ± 1,6	17	2,7 ± 1	ELISA	ng/ml	mean-sd	serum	case-control	prospectivo	33 ± 2,8	32 ± 3,2	27,2 ± 6,7	25,8 ± 5,9	No	38 ± 6	39 ± 2	0%	0%	3330 ± 162,5	3305,7 ± 165,1
Okereke et al.	2002	USA	North America	34	18,1 ± 16,2	44	10,9 ± 9,5	RIA	ng/dl	mean-sd	serum	case-control	prospectivo	N/A	N/A	N/A	N/A	Yes (32,4% GDM)	38,6 ± 1,3	39,1 ± 1,1	N/A	N/A	3480 ± 540	3400 ± 410
Aman et al.	2011	Sweden	Europe	10	17,6 ± 22,6	28	9,4 ± 14,5	RIA	mcg/L=ng/ml	median-iqr	serum	case-control	prospectivo	33,6 ± 4	29,8 ± 3,8	29,4 ± 19,1	29 ± 19,5	N/A	39,7 ± 1,1	38,8 ± 1,7	20%	35,70%	4100 ± 700	3600 ± 600
Jahan et al.	2011	India	Asia	30	40,2 ± 59,5	30	21,6 ± 35,5	ELISA	ng/ml	median-iqr	serum	case-control	prospectivo	N/A	N/A	N/A	N/A	Yes	N/A	N/A	N/A	N/A	3213,9 ± 1868,2	2842,6 ± 1401,2
Martino et al.	2016	Spain	Europe	17	40,1 ± 26,8	67	33,4 ± 40,4	ELISA	mcg/L=ng/ml	mean-sd	plasma	case-control	prospectivo	33,8 ± 4,2	30,3 ± 5,4	28,2 ± 7,5	25,5 ± 4,8	No	39,1 ± 1,3	39,3 ± 1,3	36%	21%	3392 ± 462,1	3308,1 ± 492
Shang et al.	2018	China	Asia	105	700000 ± 100000	103	600000 ± 100000	ELISA	1 mg/ml = 1000000 ng/ml	raw	plasma	case-control	prospectivo	29,8 ± 3	29,9 ± 2,9	27,8 ± 3,6	27,7 ± 3,4	Yes (9,5% GDM)	39,3 ± 1,3	39,2 ± 1	47,60%	34%	3561 ± 465	3283 ± 419
Patro-Małysza et al.	2019	Poland	Europe	26	14,9 ± 17,5	28	8,9 ± 7,1	ELISA	ng/ml	median-iqr	serum	case-control	prospectivo	35,9 ± 7,1	30,4 ± 10,9	27,3 ± 4,1	21,5 ± 3,8	Yes (100% GDM)	38,8 ± 1,4	39,7 ± 1,4	N/A	N/A	3248,2 ± 396,1	3471,4 ± 515,6
Manoharan et al.	2019	India	Asia	40	88,7 ± 28,5	40	54,7 ± 22,8	ELISA	ng/ml	mean-sd	plasma	case-control	prospectivo	24,1 ± 2,5	24,3 ± 2,2	22,1 ± 2,6	22,2 ± 1	Yes (100% GDM)	38,5 ± 1,1	39,4 ± 1,1	32,50%	27,50%	3180 ± 460	2870 ± 440
Benhalima et al.	2020	Belgium	Europe	97	12,1 ± 8,9	327	11,1 ± 9,7	RIA	mcg/L=ng/ml	median-iqr	*	case-control	prospectivo	32,3 ± 4,8	30,8 ± 3,9	N/A	N/A	Yes (13,4% GDM)	39,1 ± 1,2	39,3 ± 1,3	N/A	N/A	3408,7 ± 424,7	3349,3 ± 411,5
De Luccia et al.	2021	Brazil	South America	20	174,6 ± 130,2	38	63,5 ± 66,7	ELISA	pg/ml= 0,001 ng/ml	raw	serum	case-control	prospectivo	36,2 ± 4	32,3 ± 6,2	28,4 ± 5,1	25,2 ± 5,1	N/A	38,6 ± 0,4	39,3 ± 0,8	100% (elective)	100% (elective)	3370 ± 465,9	3338 ± 315,7
Ortega-Senovilla et al.	2022	Germany	Europe	41	17 ± 12,8	68	11,6 ± 16,6	ELISA	ng/ml	mean-SEM	serum	case-control	prospectivo	30,2 ± 5,1	28,8 ± 5,8	27 ± 5,8	25,4 ± 5,8	No	N/A	N/A	0%	0%	3410 ± 512	3500 ± 498
Anık et al.	2022	Turkey	Europe-Asia	30	8,6 ± 5,9	54	5,6 ± 5,4	ELISA	pg/ml= 0,001 ng/ml	median-iqr	serum	case-control	prospectivo	32,4 ± 4,1	32,4 ± 4,9	N/A	N/A	N/A	37,6 ± 0,8	38 ± 1,5	N/A	N/A	3526,6 ± 501,2	3158,5 ± 621

GDM, gestational diabetes mellitus; BMI, body mass index (kg/m2); N/A, not available; **gestational age at delivery (weeks); Caesarean (%); newborn weight (grams).**

4. Subgroup meta-analyses

4.1 Geographical area

Figure S1. Forest plot graphically representing the subgroup meta-analysis evaluating the changes in cord blood leptin levels between GDM patients and healthy control women, stratified by geographical area.

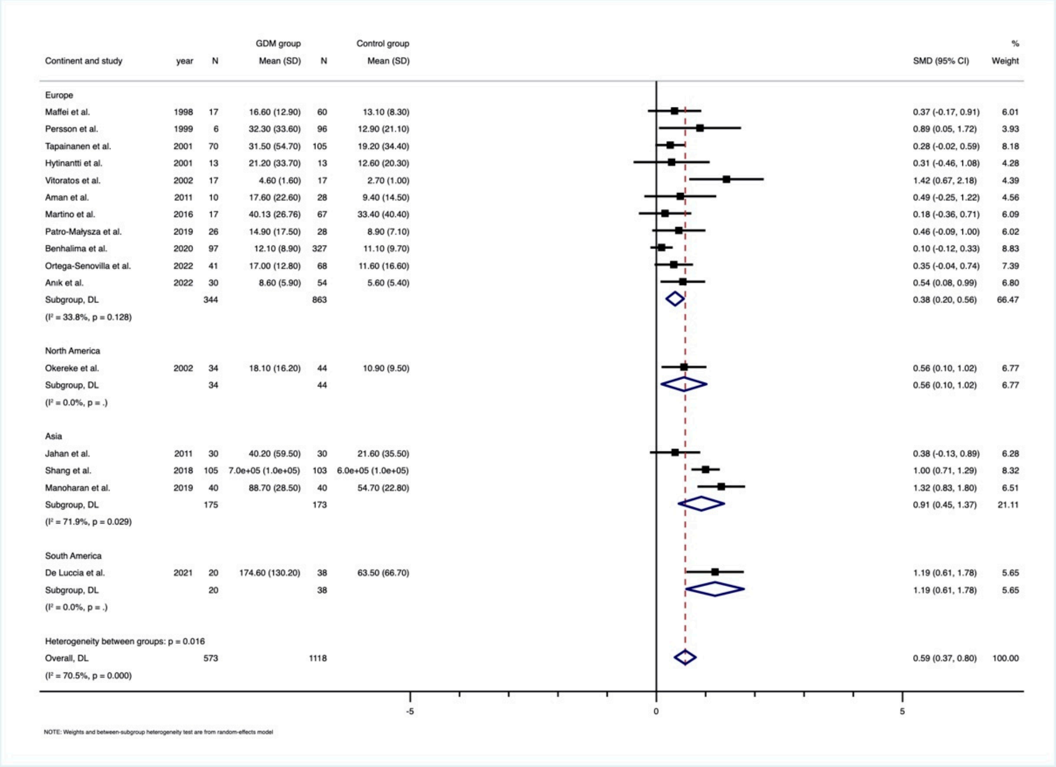

Random-effects model, inverse-variance weighting based on the DerSimonian and Laird method. Standardized mean difference (SMD) was chosen as effect size measure. A SMD>0 suggests that leptin levels are higher in GDM. Diamonds indicate the overall pooled SMDs with their corresponding 95% confidence intervals (CI).

4.2 Type of analysis technique

Figure S2. Forest plot graphically representing the subgroup meta-analysis evaluating the changes in cord blood leptin levels between GDM patients and healthy control women, stratified by type of analysis (ELISA vs RIA).

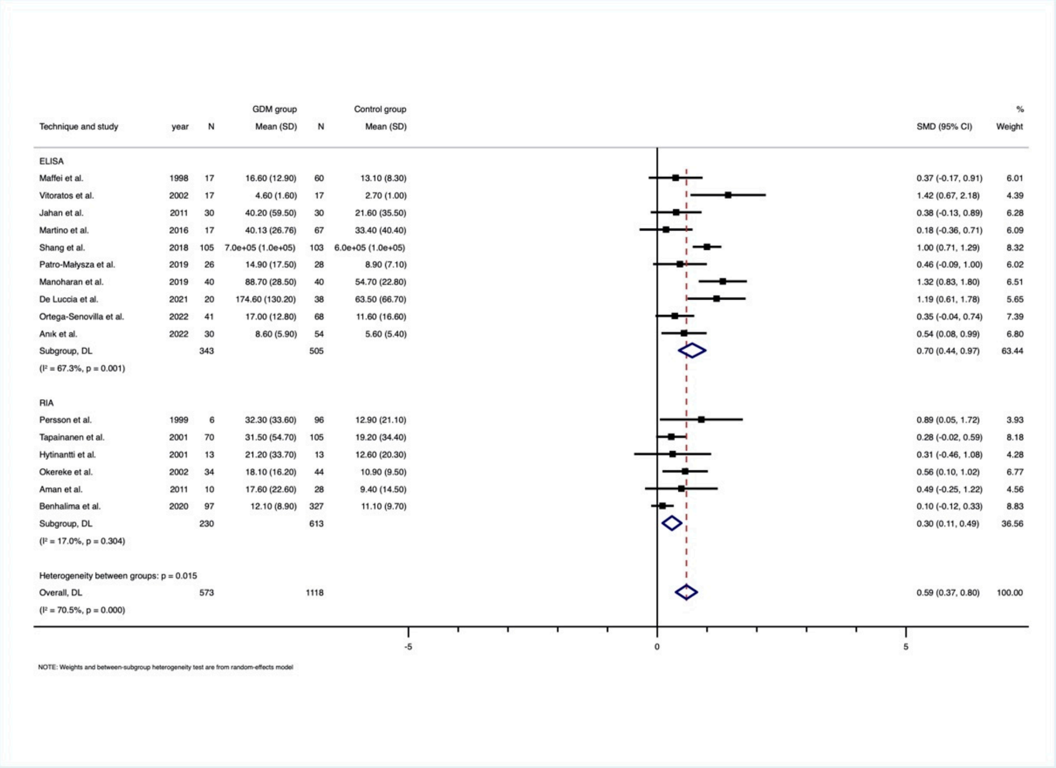

Random-effects model, inverse-variance weighting based on the DerSimonian and Laird method. Standardized mean difference (SMD) was chosen as effect size measure. A SMD>0 suggests that leptin levels are higher in GDM. Diamonds indicate the overall pooled SMDs with their corresponding 95% confidence intervals (CI).

4.3 Source of sample

Figure S3. Forest plot graphically representing the subgroup meta-analysis evaluating the changes in cord blood leptin levels between GDM patients and healthy control women, stratified by type of analysis (plasma vs serum).

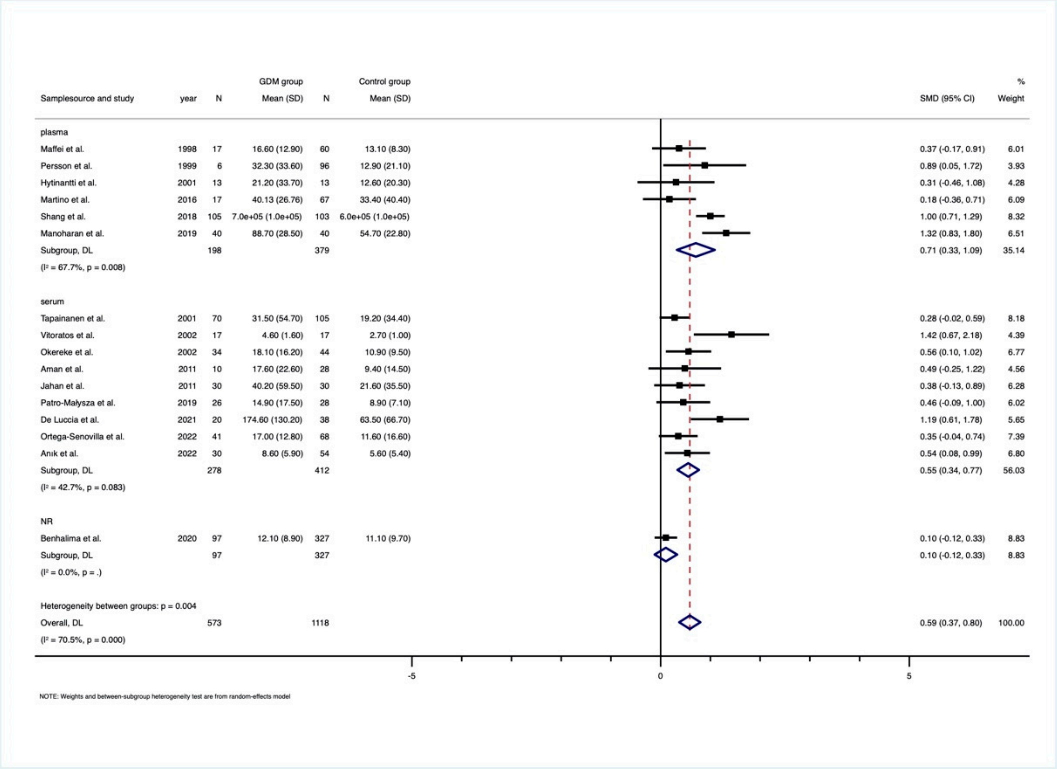

Random-effects model, inverse-variance weighting based on the DerSimonian and Laird method. Standardized mean difference (SMD) was chosen as effect size measure. A SMD>0 suggests that leptin levels are higher in GDM. Diamonds indicate the overall pooled SMDs with their corresponding 95% confidence intervals (CI).

4.4 Risk of bias

Figure S4. Forest plot graphically representing the subgroup meta-analysis evaluating the changes in cord blood leptin levels between GDM patients and healthy control women, stratified by risk of bias (low vs. moderate vs. high).

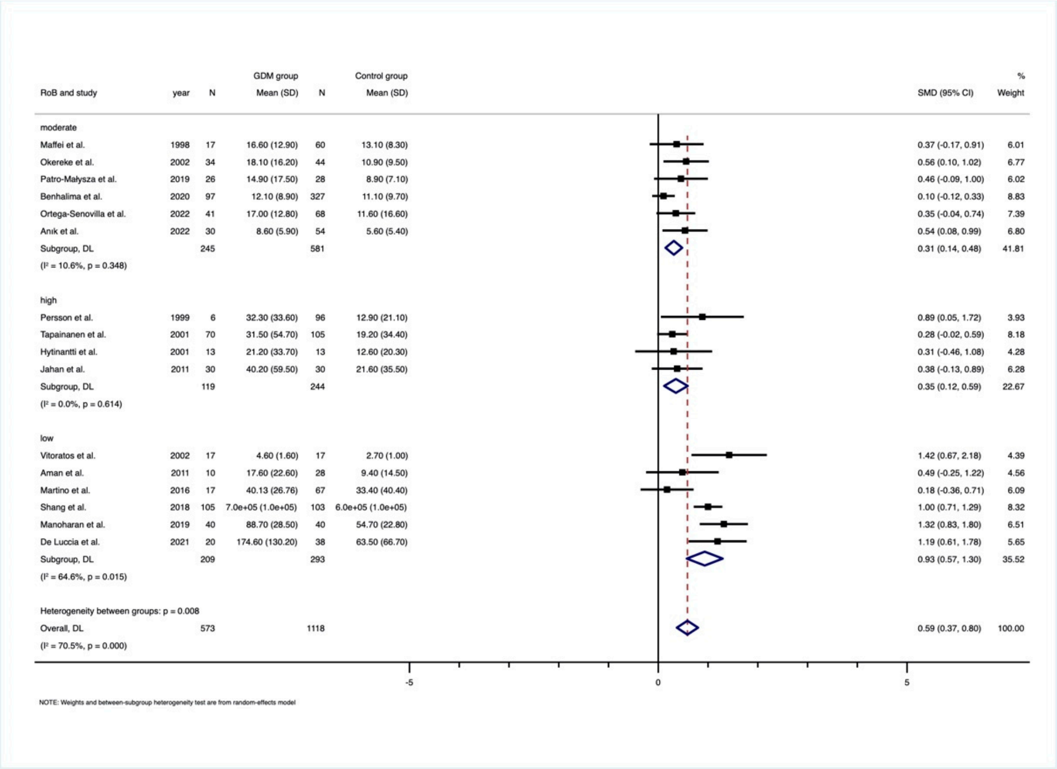

Random-effects model, inverse-variance weighting based on the DerSimonian and Laird method. Standardized mean difference (SMD) was chosen as effect size measure. A SMD>0 suggests that leptin levels are higher in GDM. Diamonds indicate the overall pooled SMDs with their corresponding 95% confidence intervals (CI).

5. Meta-regression analyses

5.1 Effect of the covariate age

Figure S5. Bubble plot graphically representing the univariable meta-regression analysis of the potential effect of age (years) on the cord blood leptin levels among patients with GDM compared with healthy control women.

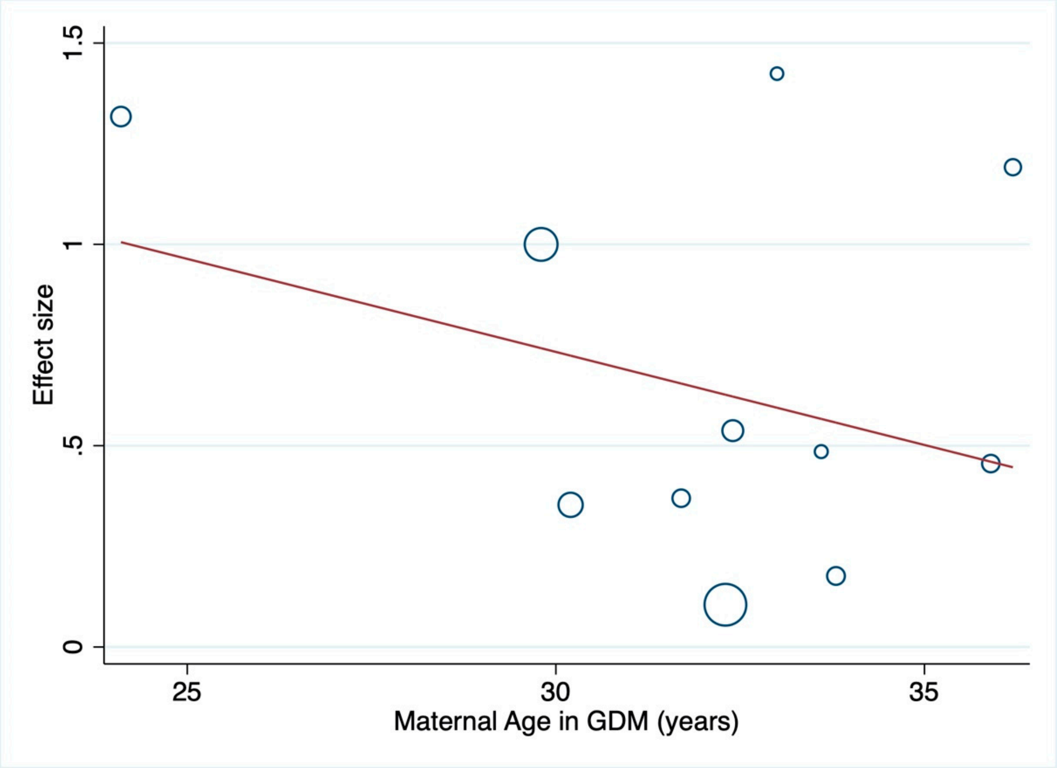

Random-effects univariable meta-regression. The red line exhibits the fitted regression line together with blue circles representing the estimates from each individual study, sized according to the precision of each estimate (the inverse of its within-study variance).

5.2 Effect of the covariate pregestational BMI

Figure S6. Bubble plot graphically representing the univariable meta-regression analysis of the potential effect of pregestational BMI (summary index score) on the cord blood leptin levels among patients with GDM compared with healthy control women.

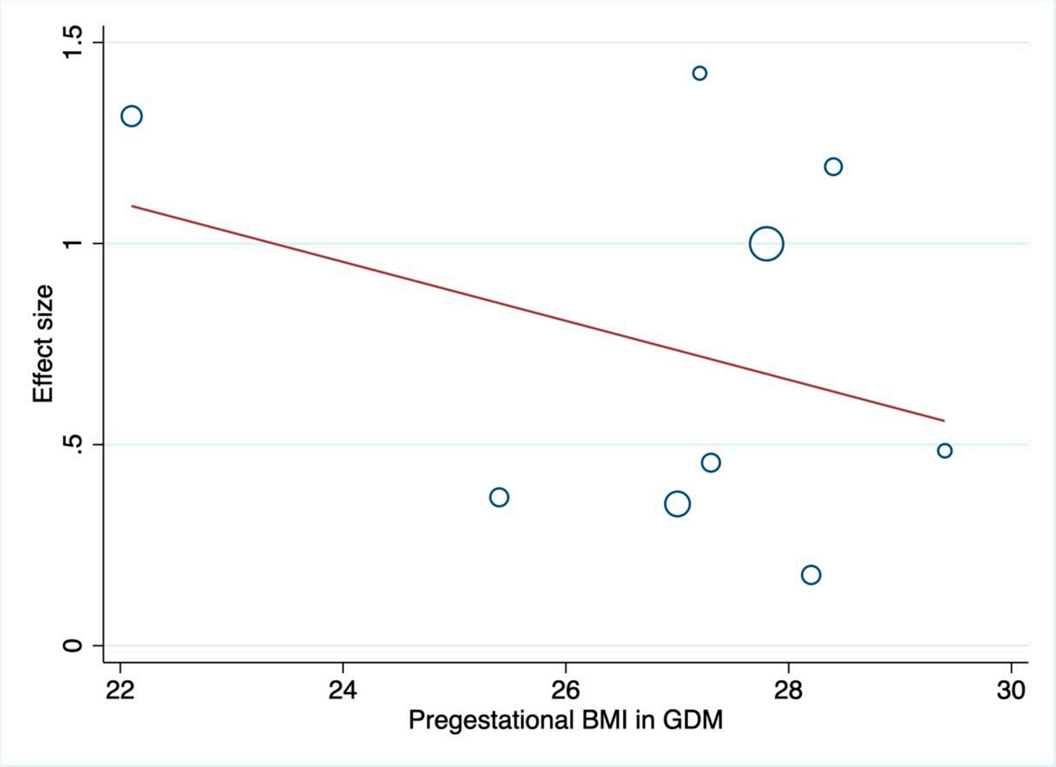

Random-effects univariable meta-regression. The red line exhibits the fitted regression line together with blue circles representing the estimates from each individual study, sized according to the precision of each estimate (the inverse of its within-study variance).

5.3 Effect of the covariate glycemia levels

Figure S7. Bubble plot graphically representing the univariable meta-regression analysis of the potential effect of glycemia levels (mmol/l) on the cord blood leptin levels among patients with GDM compared with healthy control women.

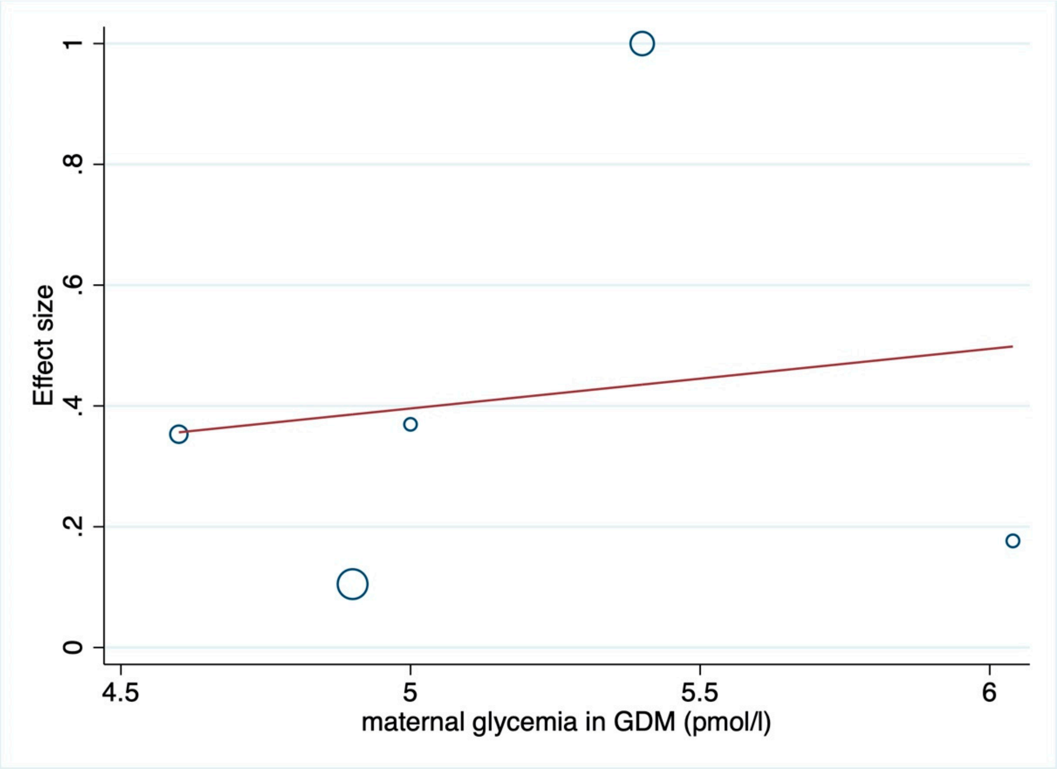

Random-effects univariable meta-regression. The red line exhibits the fitted regression line together with blue circles representing the estimates from each individual study, sized according to the precision of each estimate (the inverse of its within-study variance).

5.4 Effect of the covariate insulin

Figure S8. Bubble plot graphically representing the univariable meta-regression analysis of the potential effect of insulin (pmol/l) on the cord blood leptin levels among patients with GDM compared with healthy control women.

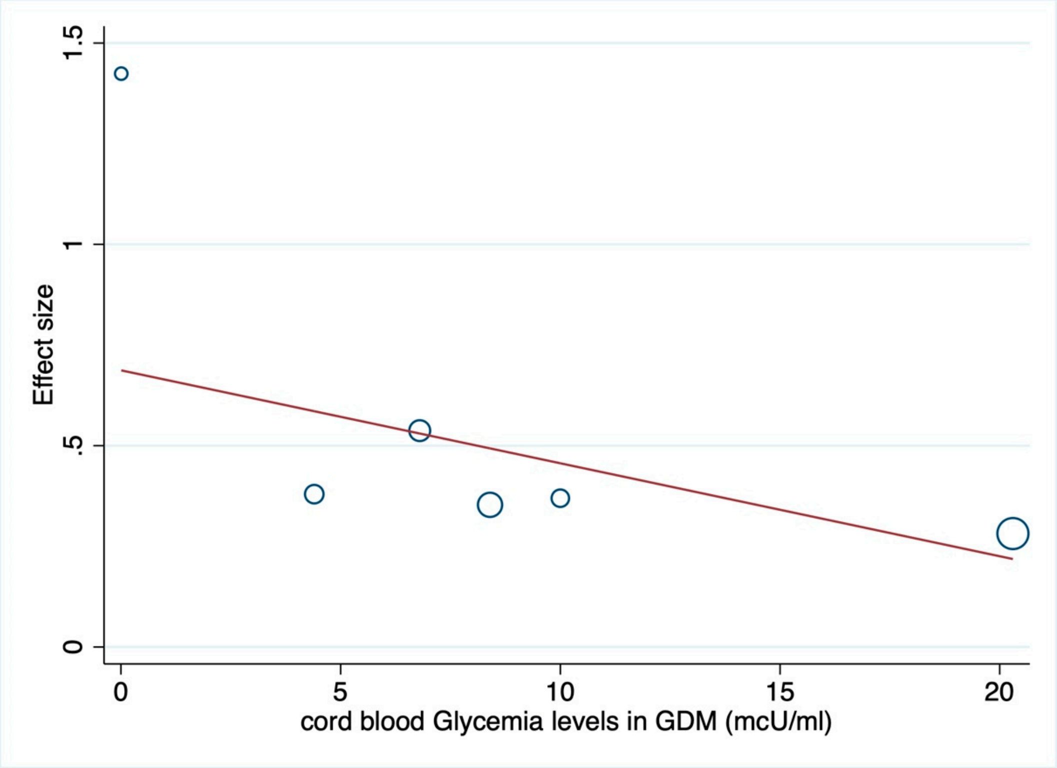

Random-effects univariable meta-regression. The red line exhibits the fitted regression line together with blue circles representing the estimates from each individual study, sized according to the precision of each estimate (the inverse of its within-study variance).

5.5 Effect of the covariate C peptide

Figure S9. Bubble plot graphically representing the univariable meta-regression analysis of the potential effect of C peptide (mmol/l) on the cord blood leptin levels among patients with GDM compared with healthy control women.

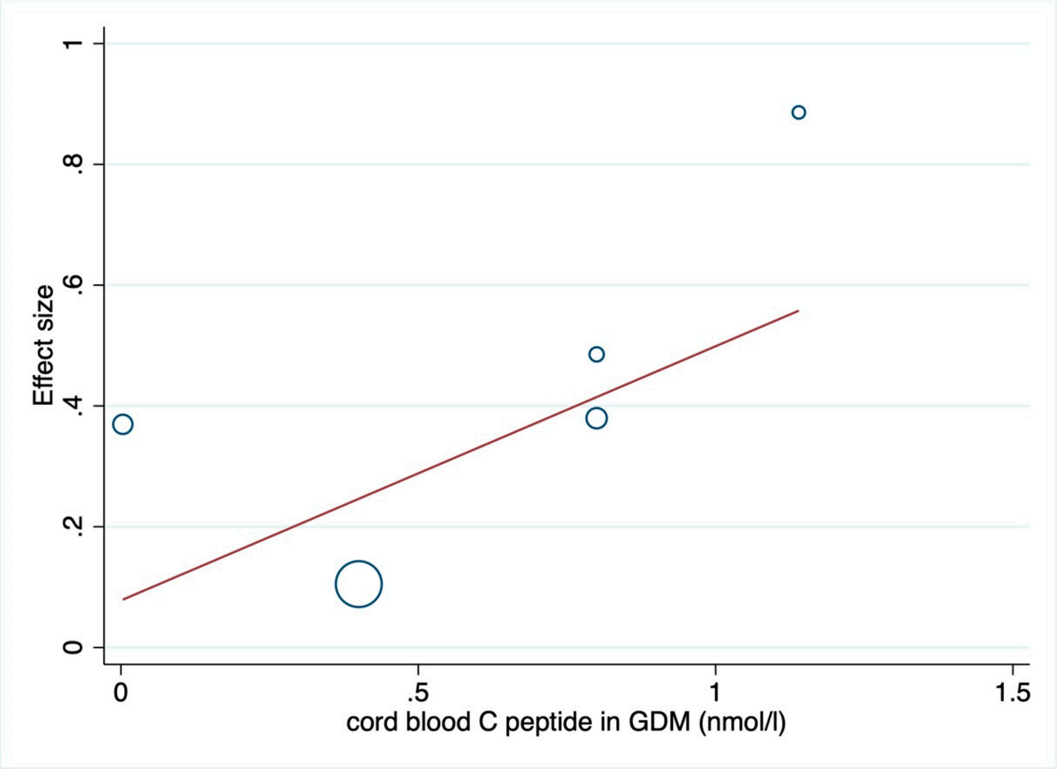

Random-effects univariable meta-regression. The red line exhibits the fitted regression line together with blue circles representing the estimates from each individual study, sized according to the precision of each estimate (the inverse of its within-study variance).

5.6 Effect of the covariate gestational age delivery

Figure S10. Bubble plot graphically representing the univariable meta-regression analysis of the potential effect of gestational age delivery (weeks) on the cord blood leptin levels among patients with GDM compared with healthy control women.

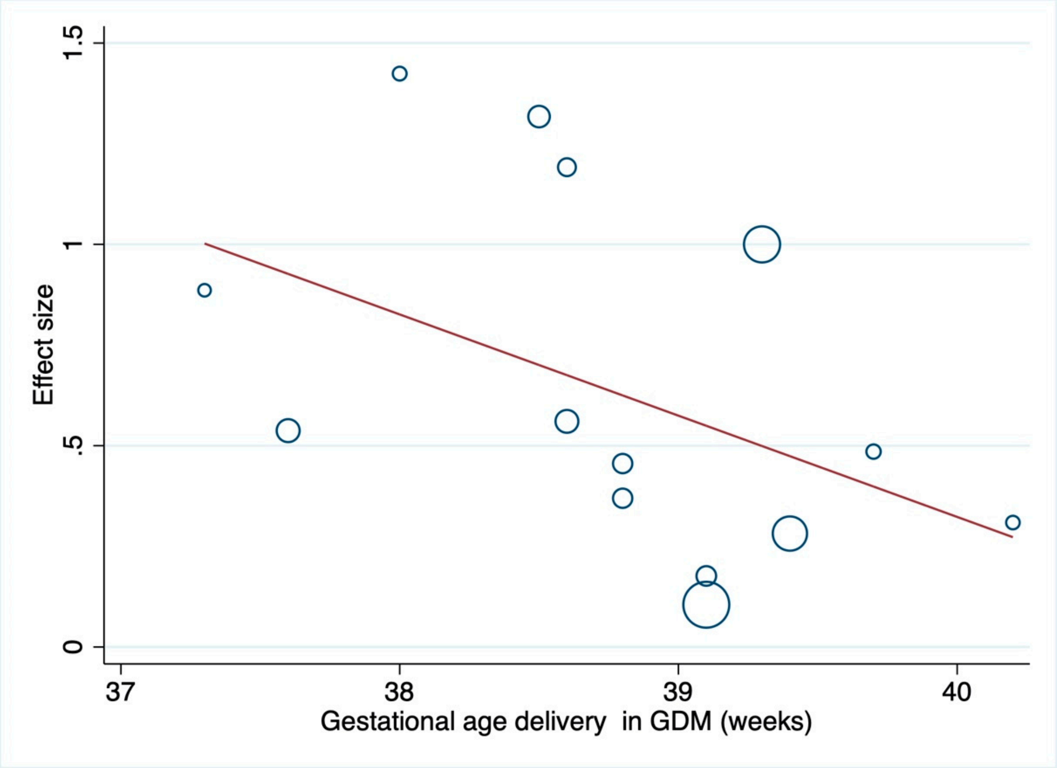

Random-effects univariable meta-regression. The red line exhibits the fitted regression line together with blue circles representing the estimates from each individual study, sized according to the precision of each estimate (the inverse of its within-study variance).

5.7 Effect of the covariate caesarian

Figure S11. Bubble plot graphically representing the univariable meta-regression analysis of the potential effect of caesarian (%) on the cord blood leptin levels among patients with GDM compared with healthy control women.

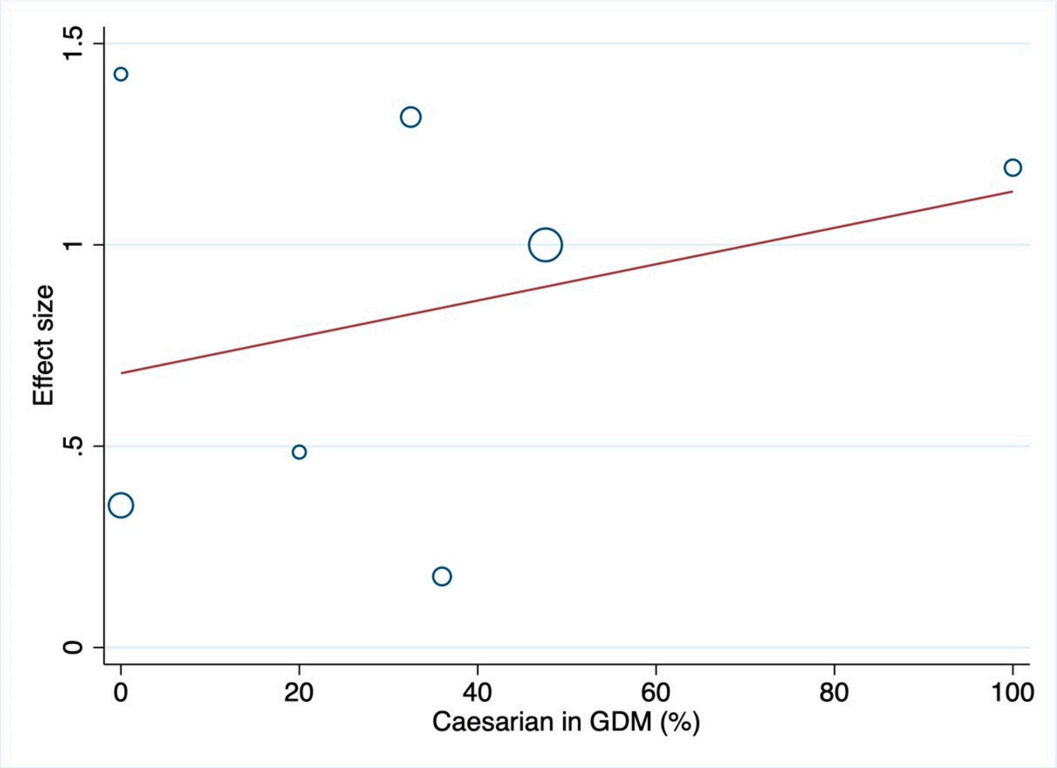

Random-effects univariable meta-regression. The red line exhibits the fitted regression line together with blue circles representing the estimates from each individual study, sized according to the precision of each estimate (the inverse of its within-study variance).

5.8 Effect of the covariate newborn weight

Figure S12. Bubble plot graphically representing the univariable meta-regression analysis of the potential effect of newborn weight (gr) on the cord blood leptin levels among patients with GDM compared with healthy control women.

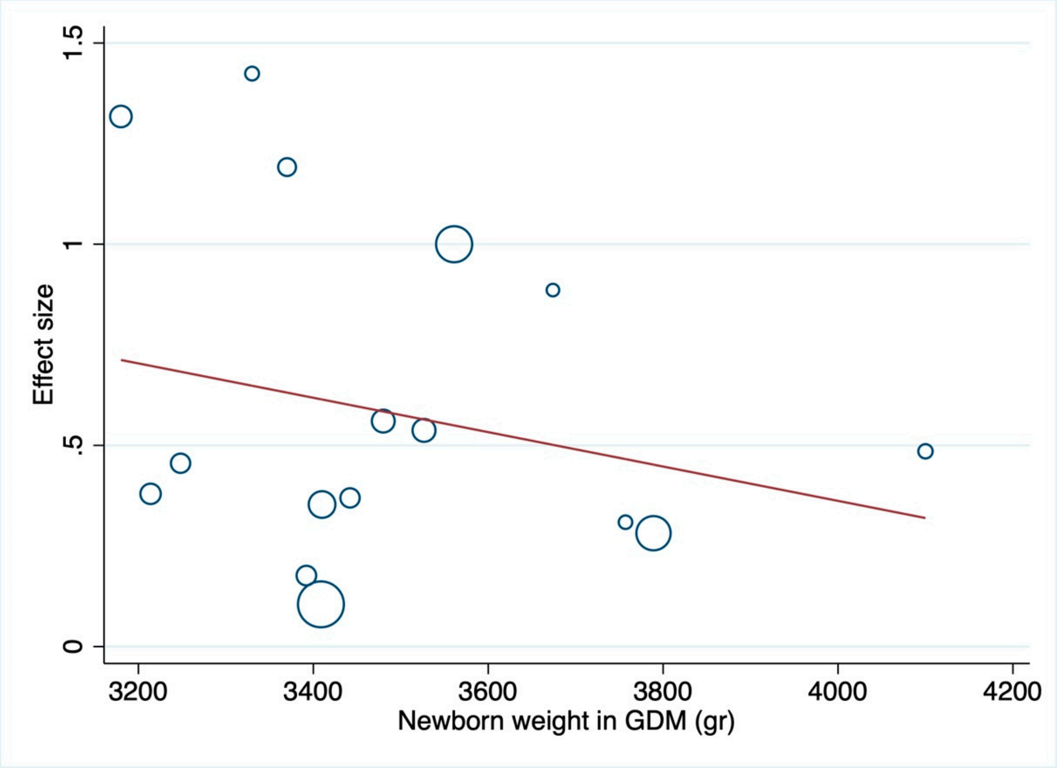

Random-effects univariable meta-regression. The red line exhibits the fitted regression line together with blue circles representing the estimates from each individual study, sized according to the precision of each estimate (the inverse of its within-study variance).

5.9 Effect of the covariate macrosomy

Figure S13. Bubble plot graphically representing the univariable meta-regression analysis of the potential effect of macrosomy (%) on the cord blood leptin levels among patients with GDM compared with healthy control women.

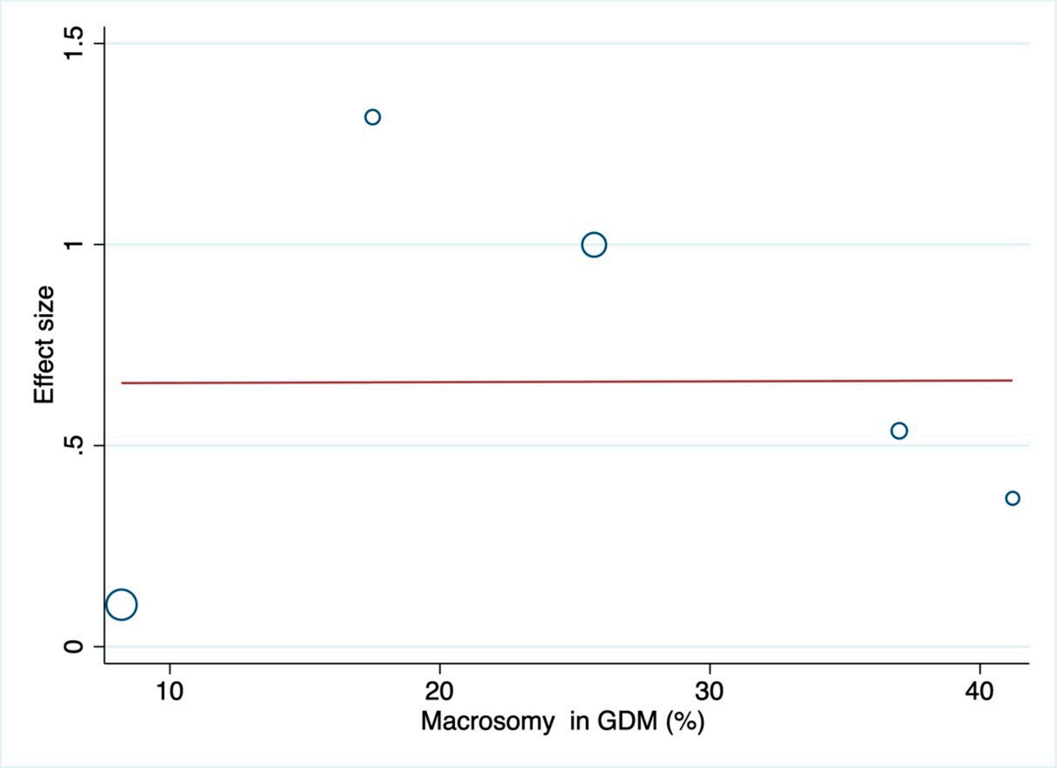

Random-effects univariable meta-regression. The red line exhibits the fitted regression line together with blue circles representing the estimates from each individual study, sized according to the precision of each estimate (the inverse of its within-study variance).

6. Sensitivity analysis.

Figure S14. Interval plot graphically representing the sensitivity analysis ("leave-one-out" method) of the studies pooled in the meta-analysis evaluating the changes in the cord blood leptin levels between GDM patients and healthy control women.

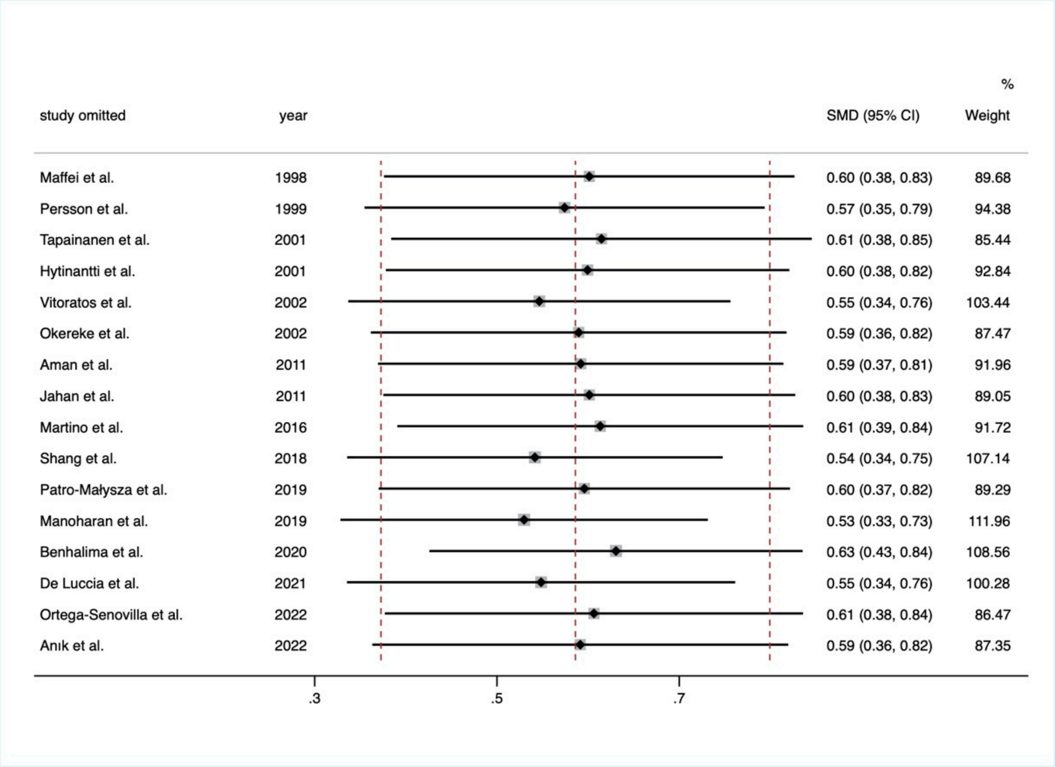

"Leave-one-out" method, sequentially omitting one study in the meta-analysis at a time, to investigate its influence on the overall result. In the interval plot, the usual diamond shape representing the pooled effect was replaced by vertical intermittent red lines, allowing a visual inspection analysis of influence.

7. Small-study effects analysis

Figure S15. Funnel plot. Funnel plots of the estimated cord blood leptin levels comparing GDM patients and healthy control women, expressed as standardized mean difference (SMD) against its standard error.

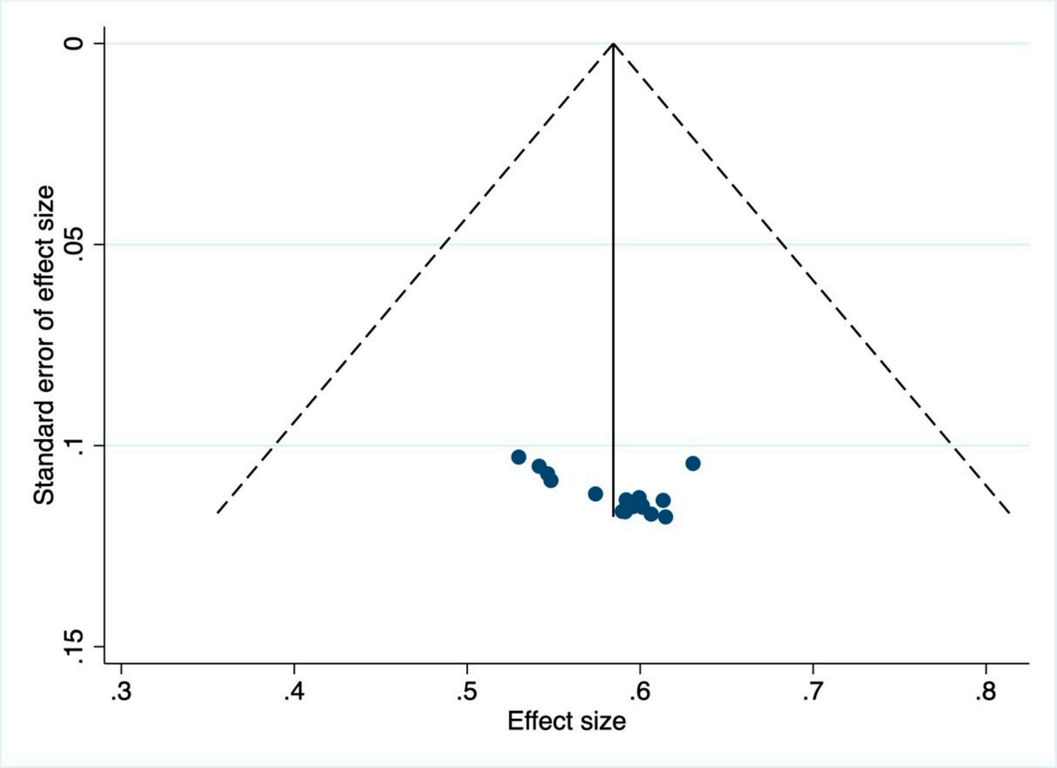

The black vertical line corresponds to the pooled SMD estimated in the meta-analysis. The two diagonal intermittent lines represent their pseudo-95%CI. The blue circles represent the estimates from primary-level studies.
